# Recent Advance of Biological Molecular Imaging Based on Lanthanide-Doped Upconversion-Luminescent Nanomaterials

**DOI:** 10.3390/nano4010129

**Published:** 2014-02-06

**Authors:** Yuanzeng Min, Jinming Li, Fang Liu, Parasuraman Padmanabhan, Edwin K. L. Yeow, Bengang Xing

**Affiliations:** 1 Division of Chemistry and Biological Chemistry, School of Physical and Mathematical Sciences, Nanyang Technological University, Singapore 637371, Singapore; E-Mails: yzmin@ntu.edu.sg (Y.M.); lijinming@ntu.edu.sg (J.L.); liuf0019@e.ntu.edu.sg (F.L.); 2 The Lee Kong Chian School of Medicine, Nanyang Technological University, 50 Nanyang Drive, Research Techno Plaza, Singapore 637553, Singapore

**Keywords:** upconversion nanoparticles, molecular imaging, luminescent nanomaterials

## Abstract

Lanthanide-doped upconversion-luminescent nanoparticles (UCNPs), which can be excited by near-infrared (NIR) laser irradiation to emit multiplex light, have been proven to be very useful for *in vitro* and *in vivo* molecular imaging studies. In comparison with the conventionally used down-conversion fluorescence imaging strategies, the NIR light excited luminescence of UCNPs displays high photostability, low cytotoxicity, little background auto-fluorescence, which allows for deep tissue penetration, making them attractive as contrast agents for biomedical imaging applications. In this review, we will mainly focus on the latest development of a new type of lanthanide-doped UCNP material and its main applications for *in vitro* and *in vivo* molecular imaging and we will also discuss the challenges and future perspectives.

## 1. Introduction

Currently, molecular imaging is a brand new and currently upcoming technology, which allows people to non-invasively monitor the cellular functions or the biological processes in complicated living systems. Usually, the observation of physiological events under single cell and/or molecular levels can provide unique insights into the function (or dysfunction) of living organisms that are normally associated with many diseases like cancer, heart disease and neurological disorders [1,2,3,4,5,6,7,8]. Therefore, molecular imaging will be a very powerful tool for its potential biomedical applications in early stage diseases diagnosis or monitoring the therapy intervention [1,2,3,4,5]. Generally, molecular imaging can be classified into different specialties that contain optical imaging, computed tomography (CT), magnetic resonance imaging (MRI), single photon emission tomography (SPECT), positron emission tomography (PET) and ultrasound imaging based on the imaging mechanism, detection instrumentation or the applied imaging contrast agents [1,2,3,4,5,6,7,8].

Among the different imaging modalities, optical imaging techniques including the fluorescence and bioluminescence imaging which utilize light sources under various wavelengths for image generation represents a simple and direct observation of specific molecular targets or biological pathways *in vitro* and *in vivo*. In general, bioluminescent imaging mainly relies on the native light emission from several organisms such as marine bacterial luciferases, the eukaryotic firefly luciferases and Renilla (sea pansy) luciferases [9,10]. Fluorescent imaging can be extensively visualized upon the light illumination of various fluorochromes with appropriate light sources and the emission signals are collected at a shifted wavelength.

Currently, quite a number of commonly used fluorochromes such as coumarin, boron-dipyrromethene (BODIPY), fluorescein, rhodamine and their analogs have been successfully applied for *in vitro* cell culture fluorescent observation and bio-sensing [11,12,13,14,15,16,17,18,19,20,21]. Some other organic fluorophores including cyanine dyes, squaraine dyes and phthalocyanines derivatives that function in the near infrared (NIR) region have also been extensively used for the non-invasively monitoring of gene expression [11], *in vivo* cell trafficking [12], enzyme activities identification [13,14,15,16,17,18,19,20,21], early stage of disease screening and new drug development [22,23] *in vitro* and in living subjects, mostly attributed to their promising advantages of reduced light scattering, high tissue penetration and relatively minimal auto-fluorescence from biological samples. However, despite the widely accepted utility of these NIR organic fluorophores in fluorescent imaging, most of the dye molecules indicated limited quantum yield, poor photostability, a short lifetime and less Stokes shift in aqueous solution [17,18,19,20,21]. Therefore, the development of alternative long emission materials with improved photo-physical properties will be still highly required.

In recent decades, the disciplines of nanomedicine and diagnostic imaging have attracted considerable academic and industry attentions, in which real-time imaging has shown to be particularly valuable for investigating the aspects, including target specificity, pharmacokinetic profiles and bio-distribution, as well as to assess the therapeutic effects of nanoparticle conjugates [15]. One of the most extensively studied nanomaterials for optical imaging applications are quantum dots (QDs) [24,25,26,27,28,29,30,31]. QDs are mono-dispersing inorganic fluorescent nano-crystalline particles made from semiconductor materials with numerous superior optical properties when compared to those of organic fluorophores [11,12,13,14,15,16,17,18,19,20,21], such as high quantum yields, high molar extinction coefficients, long fluorescent lifetimes (> 10 ns), narrow emission spectra, continuous absorption spectra ranging from the UV to NIR area and large effective Stokes shifts [24,25,26,27,28,29,30,31]. So far, QDs have attracted considerable attention in bionanotechnology and biomedical fields, particularly, multiplexed bio-sensing and labeling, targeted drug or gene delivery, and in vitro or *in vivo* biological imaging [24,25,26,27,28,29,30,31]. In spite of their significant success in principle, recent advances also highlighted the possible toxicity of Cd based QD nanomaterials in biological systems. The toxicity of the Cd related compounds would be one potential concern that limits the extensive use of these visible or NIR emitting nano-crystals, especially for their applications directly in human healthcare [27,28,29,30,31]. Therefore, searching for non-toxic, stable and more importantly, longer wavelength excited and deeper tissue penetration substitutes with high quantum yields is highly desirable and it remains a big challenge in the relevant fields.

**Figure 1:**
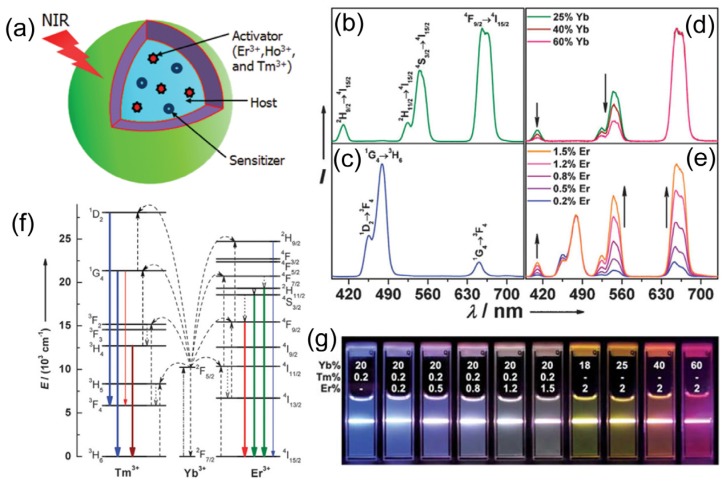
The structure and optical properties of upconversion nanoparticles (UCNPs): (**a**) Scheme illustration of the structure and components of the UCNPs; (**b**–**e**) Upconversion multicolor tuning in Ln^3+^-doped cubic NaYF_4_ UCNPs. Room temperature UCNP emission spectra of (**b**) NaYF_4_:Yb/Er (18/2 mol%), (**c**) NaYF_4_:Yb/Tm (20/0.2 mol%), (**d**) NaYF_4_:Yb/Er (25–60/2 mol%), and (**e**) NaYF_4_:Yb/Tm/Er (20/0.2/0.2–1.5 mol%) particles in ethanol solutions (10 mM). The spectra in (**d**) and (**e**) were normalized to Er^3+^ 660 nm and Tm^3+^ 480 nm emissions, respectively; (**f**) The proposed energy transfer mechanisms showing the upconversion processes in Er^3+^, Tm^3+^, and Yb^3+^ doped crystals under 980 nm diode laser excitation. The dashed-dotted, dashed, dotted and full arrows represent photon excitation, energy transfer, multiphoton relaxation, and emission processes, respectively. Only visible and NIR emissions are shown here. (**g**) Upconversion multicolor fine-tuning through the use of lanthanide-doped NaYF_4_ nanoparticles with varied dopant ratios. Adapted with permission from references [33,34,35] and [39], respectively. NIR, near-infrared. Copyright: American Chemical Society, 2008; Royal Society of Chemistry, 2009; and John Wiley and Sons, 2013.

Recently, lanthanide-doped upconversion nanoparticles (UCNPs) have been used as a primary and potentially promising vector for molecular imaging, due to their unique photo-luminescent properties and good biocompatibility. The UCNPs can absorb near-infrared (NIR) light and convert it into multiplexed emissions that span over a broad range (*i.e.*, from the UV to NIR region) [32,33], resulting in a nonlinear optical upconversion process (Figure 1), which is different from those of traditionally used down-conversion fluorescent organic fluorophores and QDs [34,35,36,37]. In general, the UCNPs indicate many advantages, including narrow-bandwidth emission, a long lifetime, tunable emission, high photostability and high resistance to photo-bleaching [38,39,40,41,42]. Furthermore, the less background auto-fluorescence and light scattering that allows deep tissue light penetration will also make UCNPs work as attractive optical contrast agents for *in vitro* and *in vivo* bio-imaging applications. More importantly, upon the doping of different elements such as Gd and Sm etc, into UCNPs structures during the process of their synthesis, the prepared UCNP can also be endowed with more promising functions for multimodality imaging by the combination of fluorescent imaging with other imaging techniques including computed tomography (CT), magnetic resonance imaging (MRI), single photon emission tomography (SPECT), positron emission tomography (PET), *etc.* [43].

So far, there are some excellent review articles that have summarized the different emphasis on the synthesis or bio-applications of UCNPs. Some mainly discussed the emphasis on synthesis [34,35], while others analyzed their applications [38,43], including the photo-physical properties of UCNPs [13,14,15,16,17,18,19,20,21,22,23,24,25,26,27,28,29,30,31]. However, reviews on the applications of molecular imaging using UCNPs are still very limited. In this review paper, we summarize the significance and recent advances in UCNPs for molecular imaging, and we also discuss the future challenges and promises of using UCNPs for molecular imaging *in vitro* and *in vivo*.

## 2. Synthesis, Surface Functionalization and Biocompatibility of Lanthanide UCNPs

In general, UCNPs are composed of a host matrix doped with a sensitizer (light absorbers) and an activator (light-emitting ions) (Figure 1) [34,35]. For activators, rare earth ions especially Ln^3+^ ions have mainly been used for activators, because of their ladder-like arranged energy level that promotes upconversion by the absorption of multiple photons. The host materials usually require close lattice matches to dopant ions, which have low phonon energies. Based on this, currently, rare earth fluorides such as binary REF_3_ and complex AREF_4_ (RE = rare earth, A = alkali), including NaYF_4_, NaGdF_4_, NaLuF_4_, KYF_4_, NaYbF_4_, LaF_3_, CaF_2_, KMnF_3_, YF_3_, KGdF_4_ and a few others are the mostly used host components for UCNP preparation [34,35,38].

Normally, the suitable size and uniform shape are the vital factors in UCNPs towards effective bio-imaging applications. There are several methods that have been developed to prepare UCNPs with appropriate size (from sub-10 nm to sub micrometer) and uniform shape (nanospheres, nanorods, nanocubes and nanoplates, *etc.*). Most of them have been prepared mainly based on techniques including thermal decomposition [33,44,45,46,47,48,49] hydrothermal synthesis [50,51,52,53,54], co-precipitation [55], sol-gel processing, *etc.* [56,57,58,59,60]. So far, among the various techniques, thermal decomposition and hydrothermal synthesis have been proven to be the two most popular methods for fabricating the UCNPs [34,35,40,41,42,43].

**Figure 2:**
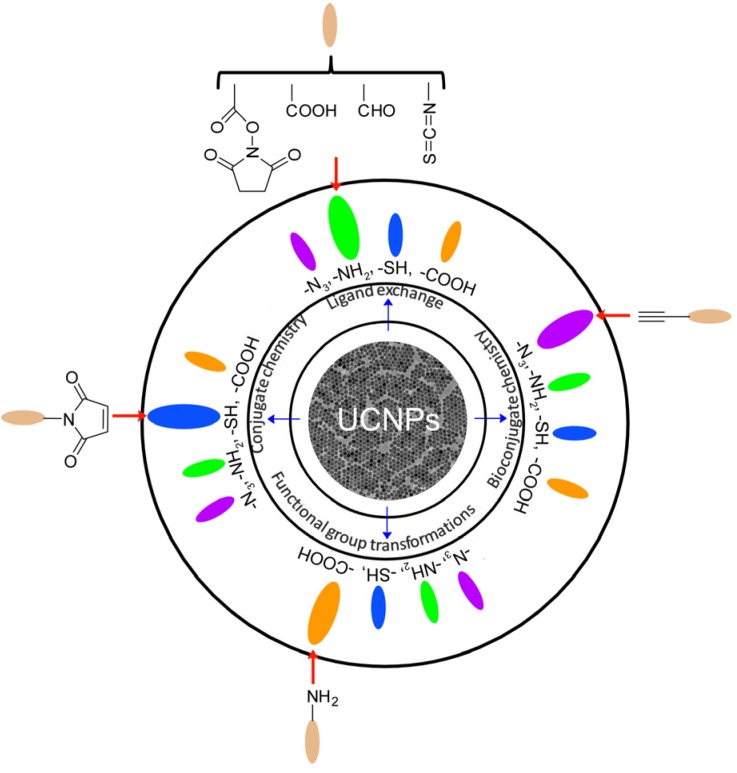
Surface functionalization of UCNPs and their functional groups for biological applications. Adapted with permission and modified from references [27,28,29,30,31]. Copyright: Royal Society of Chemistry, 2013.

Besides the size-dependent properties, the biological applications of UCNPs may also be affected by their surface properties. Currently, most of the synthesized UCNPs are usually capped with hydrophobic organic ligands, such as oleic acid (OA), oleylamine (OM) and adamantine-acetic acid (Ad), which may potentially influence their utilities in molecular imaging, mostly because of their limited solubility in aqueous solution or in biological buffers. In order to obtain biocompatible UCNPs for potential biomedical applications, it is very necessary to conduct surface modification. Therefore, water solubilization and surface functionalization are the critical steps to allow UCNP to serve as reliable platforms in biological applications. Toward this direction, a variety of strategies have been extensively reported, which mostly employ functional polymers (e.g., especially polyethylene glycol (PEG), *etc.*) [61], silica [62], peptide sequences [63] and other biomolecules [64], to functionalize UCNPs with methods of ligand exchange [65,66], ligand removal [67,68], ligand oxidation [69,70], layer-by-layer assembly [71,72], silica coating [62,73,74] or amphiphilic polymer coating, *etc.* [34,35,38,75,76]. These approaches have been successfully applied to functionalize UCNPs structures and to make them water soluble, chemical stable, biocompatible, and importantly, to have optimal reaction sites for subsequent bio-conjugation. Figure 2 illustrates the general strategies for the surface functionalization of UCNPs. It is obvious that most UCNPs have been modified with –N_3_, –NH_2_, –SH and –COOH groups, which will greatly facilitate the convenient and effective bio-conjugation on the surface of UCNPs to enhance their biocompatibility and further biomedical applications.

The biocompatibility of UCNPs is of great importance for their subsequent biological applications [77]. Therefore, the biocompatibility and potential toxicity of UCNPs need to be fully addressed before their extensive bio-applications *in vitro* and *in vivo*. So far, there has been a large amount of *in vitro* and *in vivo* experiments evaluating the cytotoxicity of UCNPs by using methods, including the MTT (methyl thiazolyl tetrazolium) assay, MTS assay (3-(4,5-dimethylthiazol-2-yl)-5-(3-carboxymethoxyphenyl)-2-(4-sulfophenyl)-2H-tetrazolium, sodium salts) and CKK-8 assays (Cell Counting Kit-8 mitochondrial metabolic activity assays) [64,78,79]. Most of these results suggested the negligible cytotoxicity of UCNPs upon the functionalization of the particle surface with different types of ligands or the aforementioned biocompatible coating molecules at cellular levels.

Furthermore, extensive *in vivo* experiments were also conducted recently to evaluate the long-term toxicity of UCNPs in different subjects such as *Caenorhabditis elegans* (*C. elegans*) worms [80], zebrafish embryos [81] and mice [64], respectively. For example, the *in vivo* results on *C. elegans* demonstrated that UCNPs prepared by homogenous precipitation in an aqueous solution of Y(NO_3_)_3_·6H_2_O (50 mM), Yb(NO_3_)_3_·5H_2_O (1 mM), Er(NO_3_)_3_·5H_2_O (0.5 mM) and urea (15 mM) did not display notable toxicity when the concentrations are up to 10 mg/mL or above [80]. The potential toxicity evaluation in mice was also conducted through behavior observation, body weight measurement, histology and hematology analysis, and serum biochemistry assay. Similar tests based on polyacrylic acid (PAA)-coated NaYF_4_:Yb/Tm UCNPs further confirmed that there was no any apparent toxicity with an administration of a dose of 15 mg/kg of PAA-UCNPs in living mice for about three months [64]. Very recently, good biocompatibility was also observed for the Gd/Mn-doped NaYF_4_:Yb/Er UCNPs which displayed very low toxicity in mice as the survival rate was 100% for thirty days after injection. Upon the analysis of inductively coupled plasma mass spectrometry (ICP-MS), it has been confirmed that Gd^3+^/Mn^2+^ ions have been confined in a rigid matrix of host UCNPs nano-crystals, which was not easily released into the living environments and, thus, greatly minimized their potential cytotoxicity [54].

## 3. Lanthanide UCNPs for Optical Imaging

In terms of superior biocompatibility and less cytotoxicity upon the surface modification, UCNPs can be used for promising biomedical applications, especially in the areas of molecular imaging, mostly owing to their unique optical properties, which can be excited with long wavelength NIR light from 795 to 980 nm, and such long wavelength light illumination can be converted into multiplexed emissions that span over a broad range from the UV to NIR region [32,33,34,35]. Such promising optical functions allow UCNP to serve as effective platforms for non-invasively visualizing biological distribution and real-time imaging of biological processes with deep tissue and light penetration.

As potential agents for molecular imaging *in vitro* and *in vivo*, UCNPs indicated many unique advantages over conventionally used organic dyes, such as narrow-bandwidth emission, a long lifetime, tunable emission, high photo-stability and high resistance to photo-bleaching. With the excitation of NIR light, the emitted light from the UV to NIR region can be applied for multicolor imaging [38,39]. Among the various converted emissions, NIR-to-NIR (using NIR excited light to emit NIR light)-based UCNPs luminescence imaging has gained increasing attention, as both the excitation and emission NIR light can penetrate the “optical transmission window” of tissues (700–1000 nm) [82], thus providing a direct powerful tool for visualizing the biodistribution of UCNPs *in vivo*.

Generally, in the process of the development of lanthanide-based UCNP nanomaterials for their applications in biological imaging, three main aspects have been classified, which include non-specific targeting imaging, active targeting imaging, and activatable imaging, mostly based on the reaction mechanism of the UCNP-based imaging probes and their related biological targets *in vitro* and *in vivo*.

**Figure 3:**
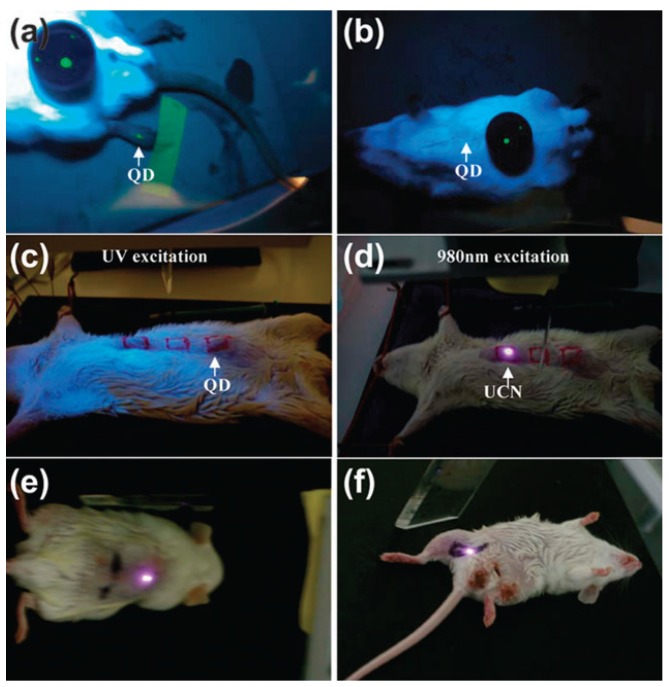
*In vivo* imaging of living rats with quantum dots (QDs) injected into the translucent skin of the foot (**a**) showing fluorescence, but not through thicker skin of the back (**b**) or abdomen (**c**), NaYF_4_:Yb/Er nanoparticles injected below the abdominal skin (**d**), thigh muscles (**e**) or below the skin of the back (**f**) show luminescence. QDs on a black disk in (**a**,**b**) are used as the control. Adapted with permission from reference [83]. Copyright: Elsevier, 2008.

**Figure 4:**
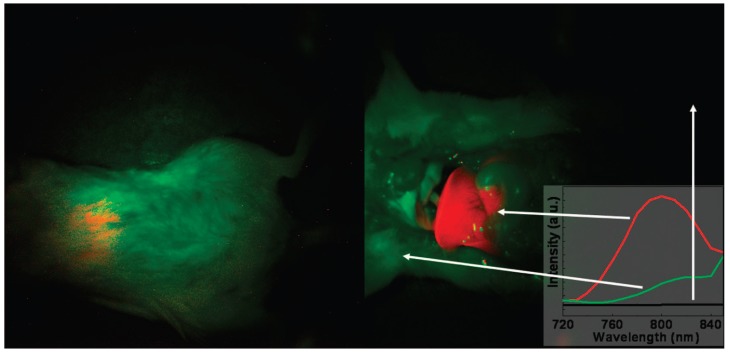
*In vivo* imaging of a mouse with the injection of UCNPs: intact mouse (**left**); the same mouse after dissection (**right**). The red color indicates emission from UCNPs; green and black show the background, as indicated by the arrows. The inset presents the photoluminescence spectra corresponding to the spectrally unmixed components of the multispectral image obtained with the Maestro system. Adapted with permission from reference [78]. Copyright: American Chemical Society, 2008.

For example, early studies of non-specific targeting imaging based on UCNPs demonstrated that 50 nm of NaYF_4_:Yb/Er UCNPs could be employed for *in vivo* imaging in anesthetized Wistar rats (Figure 3) when the rats were injected with the nanoparticles directly under the skin in the groin and upper leg regions [83]. After excitation of a 980 nm NIR laser, the nanoparticles could be detected up to 10 mm beneath the skin, far deeper than depth through the use of QDs. As one of the pioneering animal imaging studies based on UCNPs, these results provided promising examples for *in vivo* optical imaging at a deeper tissue level for performing minimal invasive imaging in real-time. With the superiority of the deep penetration of their emitted light, the NaYF_4_:Yb/Er UCNPs doped with 30 mol % Mn^2+^ ions and KMnF_3_ UCNPs doped with Yb/Er, Yb/Ho or Yb/Tm have also been applied for NIR-to-NIR single-band luminescence imaging [54]. Up to now, Tm^3+^ doped NaYF_4_, NaGdF_4_, NaLuF_4_ and NaYbF_4_ UCNPs have been extensively utilized for non-specific imaging in living mice [84,85,86]. Recently, Prasad *et al.* [78] also provided a new approach for *in vivo* imaging by using NaYF_4_:Yb/Tm-based UCNPs as high contrast photoluminescence imaging probes, with their promising light penetration depth up to ~20 mm in mouse (Figure 4).

**Figure 5:**
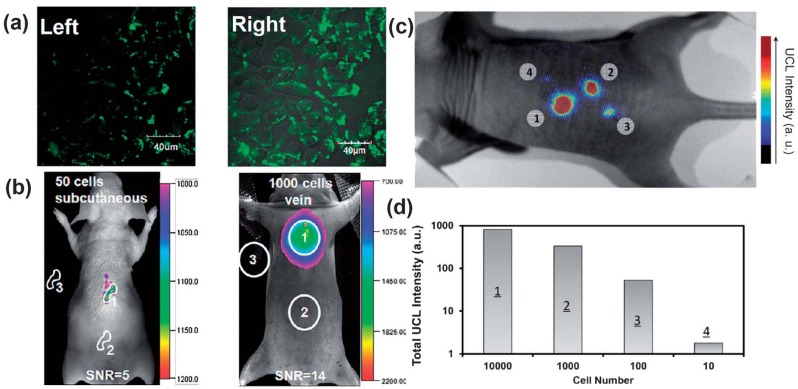
UCNPs for cellular labeling and *in vivo* tracking analysis. (**a**) Confocal UCNP imaging (**left**) and its overlay with a bright field image (**right**) of cells stained with 200 μg mL^−1^ NaLuF_4_ UCNPs for 3 h at 37 °C. (**b**) *In vivo* UCNPs imaging of athymic nude mice after subcutaneous injection of 50 human nasopharyngeal epidermal carcinoma KB cells (**left**) and tail-vein injection of 1000 KB cells (**right**). The KB cells were pre-incubated with 200μg mL^−1^ NaLuF4 UCNPs for 3 h at 37 °C before injection. (**c** and **d**) *In vivo* detection of UCNP-labeled mMSCs (an exogenous contast agent to track mouse Mesenchymal Stem Cells). (**c**) An upconversion luminescence image of a mouse subcutaneously injected with various numbers of mouse mesenchymal stem cells (1 × 10^5^) labeled with UCNPs. (**d**) Quantification of UCNPs luminescence signals in (**c**). Adapted with permission from references [87,89], respectively. UCL, upconversion luminescence. Copyright: American Chemical Society, 2011; and Elsevier, 2012.

By employing sub-10 nm citric acid-capped NaLuF_4_-based nano-crystals co-doped with Gd^3+^, Yb^3+^ and Er^3+^ (or Tm^3+^), the high contrast imaging in living mouse would allow an excellent detection limit down to 50 and 1000 UCNP-labeled cells upon the subcutaneous and intravenous injections of these nano-crystal based probes, respectively [87] (Figure 5a,b). More recently, the non-specific whole-body imaging based on polyacrylic acid (PAA) coated NaYF4:Yb/Tm (PAA-UCNPs) as luminescence probes for long-term *in vivo* distribution also showed that most of the PAA-UCNPs in the liver and the spleen would be cleared very slowly from the body through hepatobiliary excretion. Similarly, PAA-NaLuF_4_:Yb/Tm UCNPs as an optical probe have also been used in *in vivo* imaging of black mouse and even living rabbit, with an excellent signal-to-noise ratio [88]. Moreover, oligo-arginine modified UCNPs have been applied towards whole-body imaging at the single cell level using ultra-sensitive stem cell labeling for long-term stem cell tracking. It was reported that as few as ~10 cells in a mouse could be detected with an ultra-high sensitivity when compared with the currently used exogenous stem cell labeling nanoagents, such as QDs [89] (Figure 5).

Despite the promising applications of UCNPs in living animal imaging, however, as non-specific contrast agents, the biocompatible UCNPs lack reactive groups for selective recognition and further effective conjugation, which may potentially limit their extensive applications in biological imaging. Therefore, one simple and effective strategy to overcome the nonspecific targeting and to improve their selectivity towards the target site in a living system is to conjugate the bioactive ligand moieties with the UCNPs, which would exhibit strong affinity to specific molecular targets. Thus, the ligand modified UCNP conjugates can recognize the targets and can be easily trapped at the targeting region for a sufficient length of time. In the meantime, the unbound UCNPs lack affinity within the specific region and will thereby result in limited accumulation during the circulation process. In general, most of the applied high-affinity ligand moieties include small organic molecules, peptides, proteins, antibodies or their fragments [90]. Following are some representative examples for active targeting optical imaging on the basis of ligand modified UCNPs.

**Figure 6:**
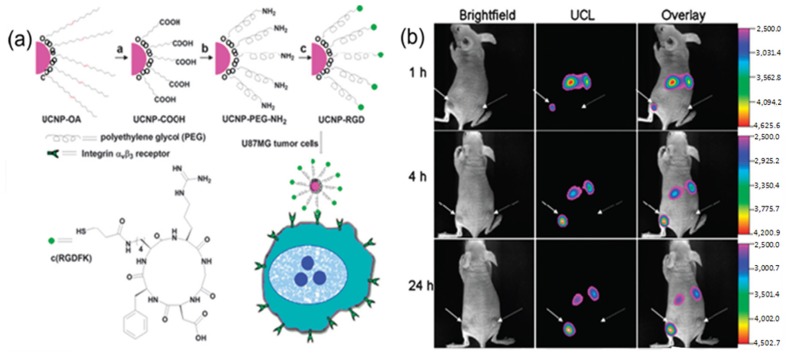
(**a**) Scheme of the synthesis of UCNP-Arginine-Glycine-Asparatic (RGD). (**b**) Time-dependent *in vivo* upconversion luminescence imaging of subcutaneous U87 MG (left hind leg, indicated by short arrows) and MCF-7 (Michigan Cancer Foundation-7) tumor (right hind leg, indicated by long arrows) borne by athymic nude mice after intravenous injection of UCNP-RGD over a 24 h period. UCNP-RGD conjugate was prepared from UCNP-OA complex (OA: Oleylamine). All images were acquired under the same instrumental conditions (power ≈ 80 mW cm^-2^ and temperature ≈ 21.5 °C on the surface of the mouse). Adapted with permission from reference [92]. Copyright: American Chemical Society, 2009.

For instance, folic acid modified UCNPs and arginine-glycine-aspartic (RGD) peptide modified UCNPs have been used for *in vivo* tumor targeting [91,92]. As indicated in Figure 6, after intravenous injection of folic acid modified UCNPs into living mouse in which tumors have been implanted by the injection of Hela cells, the Hela tumor cell lines were known to over-express folate receptors. The obvious luminescence signal at 600–700 nm was observed in the tumor, whereas no significant luminescence signal could be detected in the control group. Similarly, as revealed by other *in vivo* imaging in living animals, the RGD peptide conjugated UCNPs could also effectively target the U87 MG human glioblastoma tumor. It has been well known that U87 MG (Malignant Glioma) human glioblastoma tumors could over-express α_v_β_3_ integrin receptors, which plays an important role in tumor angiogenesis and could be easily recognized by RGD peptide sequences with high affinity [93,94,95,96]. The further *in vivo* imaging results also confirmed that the neurotoxin-mediated upconversion nanoprobes for tumor targeting and visualization in living mice could produce a high-contrast image through their highly specific binding activity toward tumors [97].

**Figure 7:**
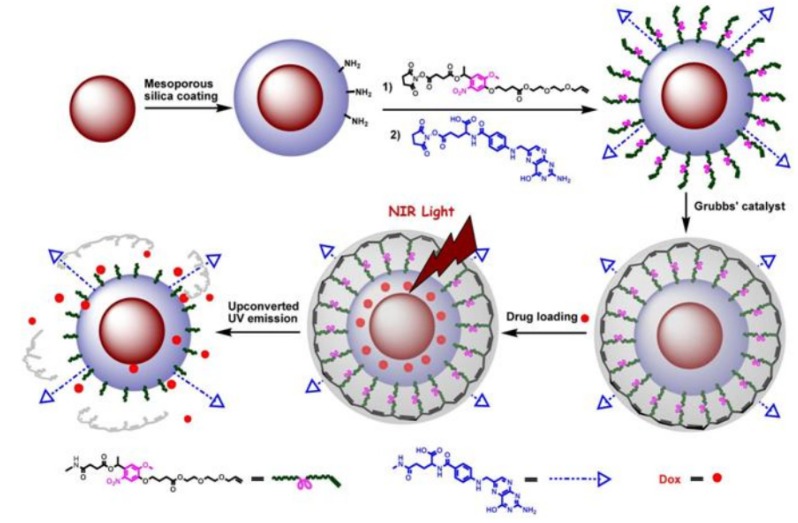
The design for photo-controlled Dox delivery through mesoporous silica coated UCNPs conjugated with folic acid. Adapted with permission from reference [73]. Copyright: John Wiley and Sons, 2013.

Similarly, upon surface modification with folic acid ligands, UCNPs could be also used as an effective platform towards targeted drug release and site specific imaging of the targeted drug release into folic acid receptor expressed HeLa cells [73] (Figure 7). By capping the mesoporous silica-UCNPs with the cross-linked *o*-nitrobenzyl photoactivatable linker, the modified silica-UCNP complex could serve as photo-caged nanocarriers to encapsulate the drug payload molecules within the mesopores silica structure. After loading with the antitumor drug, Dox (doxorubicin) followed by NIR light irradiation, the cross-linked *o*-nitrobenzyl photoactive linker on the particle surface could be efficiently cleaved by the converted UV light from UCNPs, which could effectively trigger the photo-controlled drug release into the living cells. Moreover, upon the functionalization of the photo-caged nano-carriers with folic acid units, selective drug delivery can be easily achieved in the targeted tumor cell lines in which the folate receptor has been highly expressed. This novel and effective drug loaded photocaged nanocarrier may demonstrate a new possibility for selective cell imaging and controlled drug release in the living system with less photo damage and deeper light penetration.

In line with the selective recognition of the biological imaging targets in living system, one more alternative strategy to effectively impart the molecular specificity into activatable imaging contrast agents is to develop environmentally responsive targeting probes, which would rely on the controlled manipulation of the fluorescent output of UCNP platforms by changing their local chemical environments or structural conformations. On the basis of this process, the fluorescent signal could be generated or significantly amplified only after the particle-based nano-probes have been specifically activated by an environmental stimulation.

Among the different strategies to respond to environmental stimulation, photolysis of photoactivatable or “caged” molecules has been well proven to be one effective strategy for the non-invasive regulation of biological activities and processes in living systems [98,99,100,101], by which the activation process could be easily modulated by a beam of light with high spatial and temporal precision. Generally, in this process, the active sites in targeted biological molecules were covalently modified with photo-labile protecting groups (e.g., 2-nitrobenzyl derivatives, spiropyran or diazobenzene moieties, *etc.*), which would keep these molecules at inactive stages before photolysis. Upon brief light irradiation, these photo-active protecting groups could be cleaved and the photo-caged biological molecules would be released, thus recovering the intrinsic bioactivity [98,99,100,101]. So far, the photo-caged activation strategies have been successfully applied as powerful tools to monitor biological processes and study their native functions in well-resolved space and time resolutions. However, up to now, most of photoactivatable compounds have been designed to mainly respond to UV light irradiation and they could not be activated by visible or NIR light. In general, there were significant shortcomings associated with the utilities of the short wavelength of UV or visible light in the process of photoactivation. For example, excessive exposure to UV light would easily lead to photo cross-linking reactions in nucleic acids or proteins, which could induce significant cellular damage. Meanwhile, short-wavelength UV or visible light irradiation could not penetrate into deep tissue, which greatly limited their extensive applications for living animal imaging and the activation of the photo-caged biomolecules in living conditions. Therefore, the rational design of new photo-caged strategies or alternative materials for high depth of tissue or light penetration but with limited biological damage would be of utmost importance. Herein, by taking the advantage of the unique properties of UCNPs to transmit NIR to UV or visible light emission, lanthanide-doped particle systems have received considerable attention and they could work as promising functional materials towards photo-activatable imaging *in vitro* and *in vivo* without worrying about the issues caused by direct UV or visible light irradiation.

**Figure 8:**
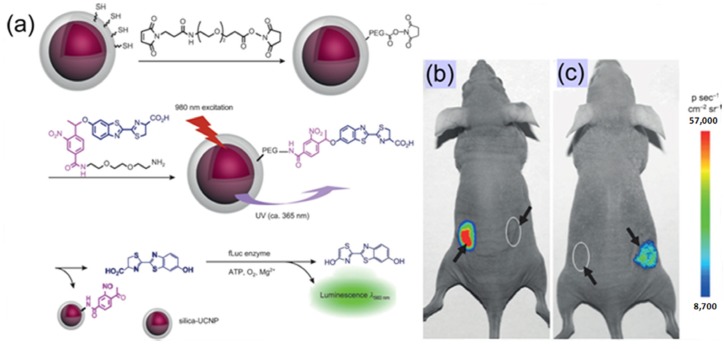
Bioluminescent images of firefly luciferase (fLuc) activity in living mice that were treated with D-luciferin. (**a**) Experimental design for uncaging D-luciferin and subsequent bioluminescence through the use of photo-caged core-shell upconversion nanoparticles. (**b**) **Left**: injection with D-luciferin (20 μM, 20 μL); **right**: injection with photo-caged nanoparticles without NIR light irradiation. (**c**) **Left**: injection with photo-caged nanoparticles and irradiation with UV light for 10 min; **right**: injection with photo-caged nanoparticles and irradiation with NIR light for 1 h. Adapted with permission from reference [62]. Copyright: John Wiley and Sons, 2012.

As proof-of-concept, Xing’s group [62] recently reported a simple and effective method for the controlled uncaging of D-luciferin and bioluminescence imaging on the basis of photo-caged UCNPs, which takes advantage of the photon upconversion of NIR light to UV light to trigger the uncaging of D-luciferin from D-luciferin-conjugated UCNPs (Figure 8). As the first example of photo-caged imaging through UCNPs, the released D-luciferin effectively conferred enhanced fluorescence and bioluminescence signals *in vitro* and *in vivo* with deep light penetration and low cellular damage.

**Figure 9:**
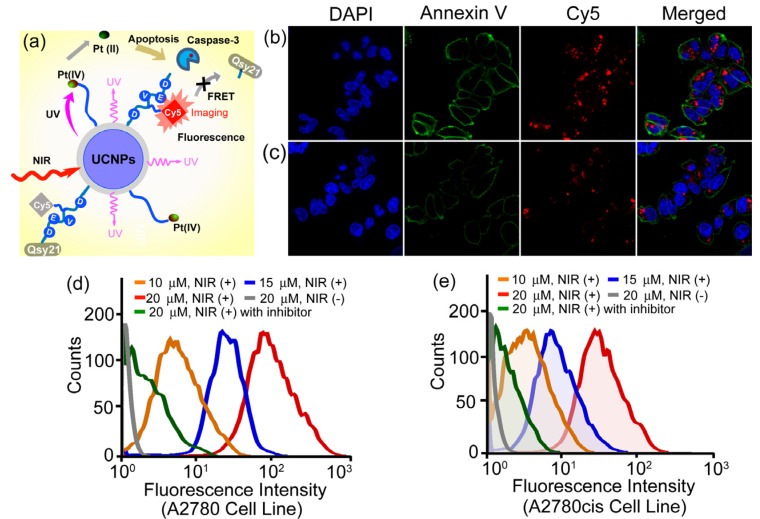
Live-cell apoptosis imaging for NIR irradiation of Pt(IV)-probe UCNPs@SiO_2_ (15 µM) incubated cells: (**a**) schematic illustration of NIR light activation of platinum(IV) prodrug and intracellular apoptosis imaging through upconversion nanoparticles; (**b**) A2780 cells; and (**c**) cisplatin resistant A2780cis cells. (blue: DAPI (4,6-Diamidino-2-phenylindole); green: Annexin V; red: Cy5.) Quantitative flow cytometric analysis of (**d**) A2780 and (**e**) A2780cis cells treated with different concentrations of Pt(IV)-probe UCNPs@SiO_2_ (10, 15 and 20 µM, respectively) and 1 h NIR irradiation. Cells treated with Ac-DEVD (Aspartic acid-Glutamic acid-Valine-Aspartic acid)-CHO (20 µM) inhibitor and NIR irradiation of cells without Pt(IV)-probe UCNP incubation were used as controls. Adapted with permission from reference [102]. Copyright: John Wiley and Sons, 2013.

More recently, the same group also demonstrated a novel and personalized near-infrared (NIR) light activated prodrug nano-platform through the combination of photoactivatable platinum(IV) prodrug and caspase imaging peptide conjugate with silica coated upconversion nanoparticles (UCNPs) for the remote control of antitumor platinum prodrug activation and simultaneously, for the real-time imaging of apoptosis induced by the activated cytotoxicity [102] (Figure 9). Upon NIR light illumination, the Pt(IV) prodrug complex could be activated from the surface of nanoparticles and the selective release of active component could exhibit potent cytotoxicities against human ovarian carcinoma A2780 cells and their cisplatin resistant variant A2780cis cells. More importantly, the systematic programmed cell death was also found to be involved in the NIR responsive tumor inactivation, and the triggered caspases enzymes would effectively cleave the recognition peptide sequence with a flanking fluorescent resonance energy transfer (FRET) pair consisting of NIR fluorophore, Cy5 and relevant quencher Qsy21, thereby allowing the direct imaging of apoptosis by separating Cy5 from Qsy21 quencher *in vitro* and in living cells. Such specific and novel rational design exhibited the promising opportunity to remotely control the activation of platinum drug in the targeted tumors; meanwhile, it could also evaluate in real time the antitumor activities by imaging of apoptosis triggered from the photoactivated drug release, thus providing great potentials for personalized tumor therapeutic and diagnostic applications on the basis of UCNPs in the future.

## 4. Doped Lanthanide UCNPs for Multimodality Imaging

Apart from applications in optical imaging, UCNPs could be further developed to incorporate multiple modality imaging moieties towards integrated identification of specific biological pathways and anatomic structures under high-resolution and three dimensional resolutions, as well as real-time monitoring of physiological functions in living tissues. Upon the structural doping or labeling of bio-medically applied elements, such as Gd, ^18^F, *etc.* UCNPs could be extensively devised to expand their further applications in multifunctional imaging not only through individual MRI (Magnetic Resonance Imaging), PET (Photoinduced Electron Transfer), CT (Computed Tomography), and SPECT (Single-Photon Emission Tomography) imaging but also through processes based on dual-modal or multi-modal imaging techniques.

For example, Li *et al.* [84] reported Gd-doped UCNPs that could offer both T_1_-weighted MRI contrast and optical imaging for *in vivo* dual-modal imaging in living mice. Besides the Gd-doped UCNPs, super-paramagnetic iron oxide nanoparticles (IONP) have been also modified to integrate into hetero-UCNPs structures by surface shielding with a silica shell to achieve dual-modal T_2_-weighted MRI and fluorescent imaging through one unified platform. Recently, Liu *et al*. [103] also developed a novel type of multifunctional nanoparticles based on UCNP@IONP@Au with combined optical and magnetic properties useful in dual-modal imaging and targeted photo-thermal tumor therapy in living subjects.

**Figure 10:**
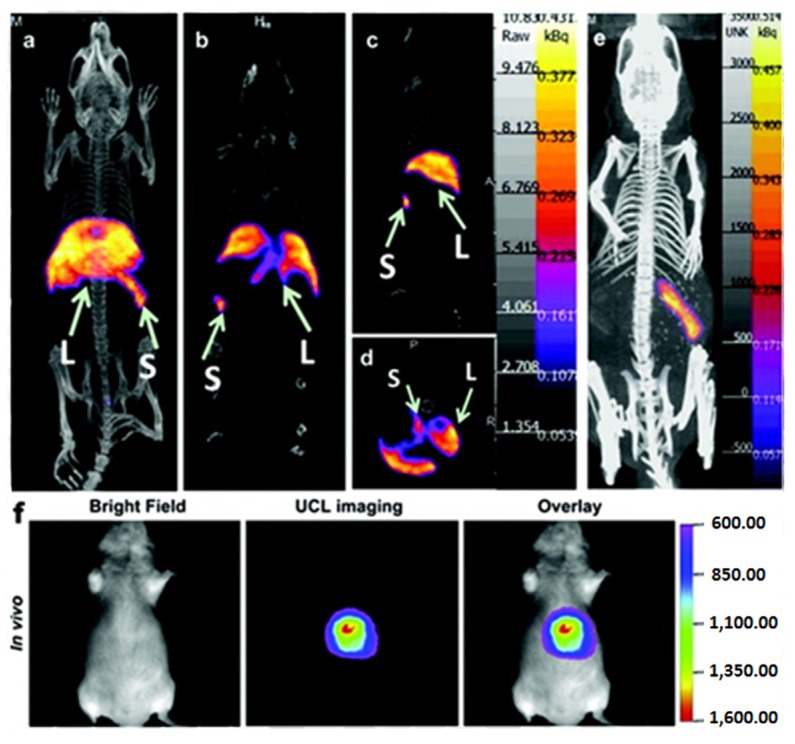
*In vivo* Single Photon Emission Tomography (SPECT)/Optical imaging study after intravenous injection of ^153^Sm-UCNPs. (**a**) Whole-body three-dimensional projection, (**b**) coronal, (**c**) sagittal and (**d**) transversal images acquired at 1 h and (**e**) whole-body three-dimensional projection images acquired at 24 h are shown respectively. The arrows in the inset point to the liver (L) and spleen (S). (**f**) *In vivo* upconversion luminescence imaging of the Kunming mouse 1 h after tail vein injection of the ^153^Sm-UCNPs (20 mg/kg). Adapted with permission from reference [105]. Copyright: Elsevier, 2013.

Dual-modal CT/optical imaging *in vivo* was also reported using lanthanide-doped NaGdF_4_ UCNPs as effective contrast agents with both of the signals co-located well in the subcutaneous injection site in living mice [104]. Very recently, Li *et al.* [105] reported ^18^F-labeled NaYF_4_:Yb/Tm UCNPs for PET and luminescent imaging of sentinel lymph node in different living animals. In this study, the UCNPs could be tracked in real time with high sensitivity *in vivo* from mice to large animals by the designed dual modality X-ray and fluorescent imaging strategies. Moreover, considering the potential issue of the short half-life of ^18^F, the same group also developed another ^153^Sm-labeled NaLuF_4_:Yb/Tm UCNP through the hydrothermal synthesis strategy for improved SPECT and fluorescent imaging of living animals. In this new system, the half-life of ^153^Sm (46.3 h) was found to be greatly enhanced [106], and the biological distribution of the ^153^Sm-labeled NaLuF_4_:Yb/Tm UCNPs had been well quantified *in vivo* using the SPECT imaging technique which was consistent with the method done by fluorescent imaging (Figure 10).

**Figure 11:**
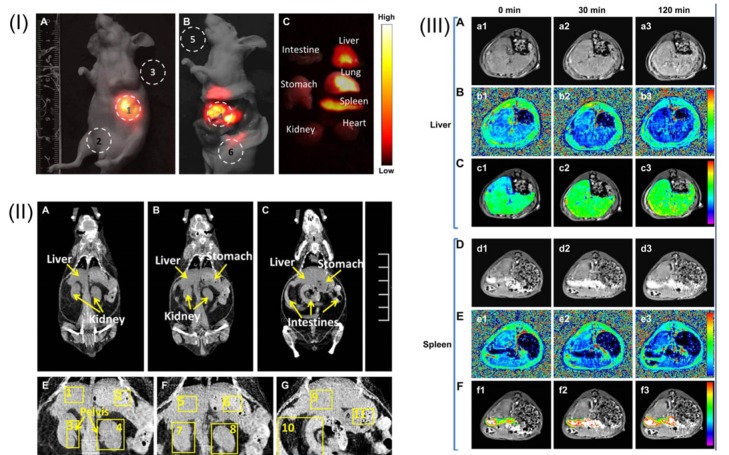
(**I**) A whole-body imaging of UCNPs@SiO_2_-GdDTPA (Diethylenetriaminepentaacetic Acid) for 10 min. (**I-A**) *In vivo* imaging of the sacrificed nude mouse after injection with UCNPs@SiO_2_-GdDTPA for 10 min. (**I-B**) *Ex vivo* imaging of nude mouse. (**I-C**) *Ex vivo* imaging of viscera. All images were acquired under the same instrumental conditions, and the power density of the 980 nm laser is 150 mW cm^−2^. (**II**) The application of *in vivo* CT imaging in Kunming mice. (**II-A**, **B** and **C**) serials coronal CT images of Kunming mouse at different layer after injection with UCNPs@SiO_2_-GdDTPA. (**II-E**, **F** and **G**) partial enlarged CT view of abdomen. (**III**) The application of *in vivo* MRI imaging of the Kunming mice. (**III-A**) T_1_-weighted MR images of liver after injection with UCNPs@SiO_2_-GdDTPA for 0, 30 and 120 min (**III-B**) T_1_ distribution images of liver after injection with UCNPs@SiO_2_-GdDTPA for 0, 30 and 120 min. (**III-C**) Local colorized T_1_-weighted MR images of liver after injection with UCNPs@SiO_2_-GdDTPA for 0, 30 and 120 min. (**III-D**) T_1_-weighted MR images of spleen after injection with UCNPs@SiO_2_-GdDTPA for 0, 30 and 120 min. (**III-E**) T_1_ distribution images of spleen after injection with UCNPs**@**SiO_2_-GdDTPA for 0, 30 and 120 min. (**III-F**) Local colorized T_1_-weighted MR images of spleen after injection with UCNPs@SiO_2_-GdDTPA for 0, 30 and 120 min. Adapted with permission from reference [85]. Copyright: Elsevier, 2012.

Furthermore, besides the dual-modal imaging based on doped UCNPs, multi-modal imaging through the doping of the different elements into lanthanide-based UCNPs structures could be also achieved. For example, the fabrication of ^18^F-labeled NaGdF_4_:Yb/Er UCNPs have been reported which have been further used for PET/MRI/optical triple-modal imaging *in vitro* and *in vivo* [107]. Similarly, the combination of Gd-doped UCNPs with gold nanoparticles based on a simple electrostatic adsorption mechanism was also reported recently and such a sub-50 nm sized multifunctional nanostructure has been proven to be useful for CT, MRI and fluorescent imaging in living mice [108]. A similar triple-modal imaging application was also reported on the basis of Gd^3+^ complex-modified NaLuF_4_ core-shelled upconversion nanophosphors for CT, T_1_-enhanced MRI and NIR-to-NIR fluorescent imaging of tumor status in living subjects [85] (Figure 11); moreover, the MTT assay and histological analysis of viscera sections further confirmed the good biocompatibility and minimum biological toxicity of mixed UCNPs@SiO_2_-Gd-DTPA (Diethylenetriaminepentaacetic Acid) nanoparticle components *in vitro* and in the living animals.

## 5. Summary and Perspectives

This review summarizes the latest advance of lanthanide doped UCNPs within the past few years, mainly concentrating on their bioapplication in molecular imaging *in vitro* and in living subjects. Indeed, upon effective surface modification and light irradiation under long wavelength (mostly at 980 nm), UCNPs particles have been proven to be highly beneficial for real-time imaging of biological processes with a high depth of tissue penetration and, meanwhile, with minimum cellular damage in living systems. Moreover, by taking advantages of the unique luminescent and chemical properties of UCNPs, such promising functional materials will also open new possibilities for more extensive applications, including medical diagnostics and imaging, targeted drug or gene delivery, and light controlled therapy in the future. So far, multifunctional nanoprobes based on UCNPs are emerging as a new type of theranostic tool to achieve more accurate diagnostics and more effective treatments through the integrated multimodal imaging moieties towards MRI, CT, SPECT, PET and photoacoustic screening [84,103,104,105,106,107,108]. Similarly, novel multifunctional materials by the combination of photo-activated therapeutic agents with UCNP nanostructures have also been proposed recently to investigate the possibility for improved *in vivo* therapeutics based on the different strategies including siRNA treatment, photodynamic therapy (PDT), and photothermal therapy (PTT), *etc.* [32,42,109,110,111,112]. Toward these ends, by right, outstanding and promising progress of great scope has been made in designing new types of functional UCNPs and these studies definitely enhance the collaborative efforts from scientists with expertise in diverse research areas. However, despite the great potentials of UCNPs in biomedical applications, many challenges certainly remain to be fully addressed and substantial efforts still need to be made to solve the problems in the applications of lanthanide-based UCNPs.

**Figure 12:**
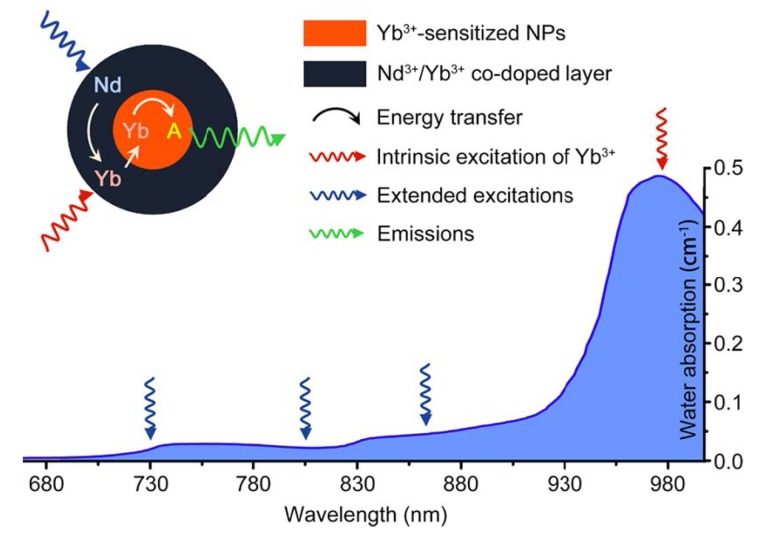
Absorption of water in the NIR and the integration scheme of the Nd^3+^→Yb^3+^ energy transfer (ET) process by introducing the Nd^3+^/Yb^3+^ co-doped shell. The resulting Nd^3+^→Yb^3+^→activator ET could extend the effective excitation bands for conventional Yb^3+^-sensitized UCNPs. Featuring lower water absorptions, these alternative excitation bands are expected to minimize the tissue overheating effect caused by NIR laser exposure (the blue line represents the absorption spectrum of water). Adapted with permission from reference [113]. Copyright: American Chemical Society, 2013.

**Figure 13:**
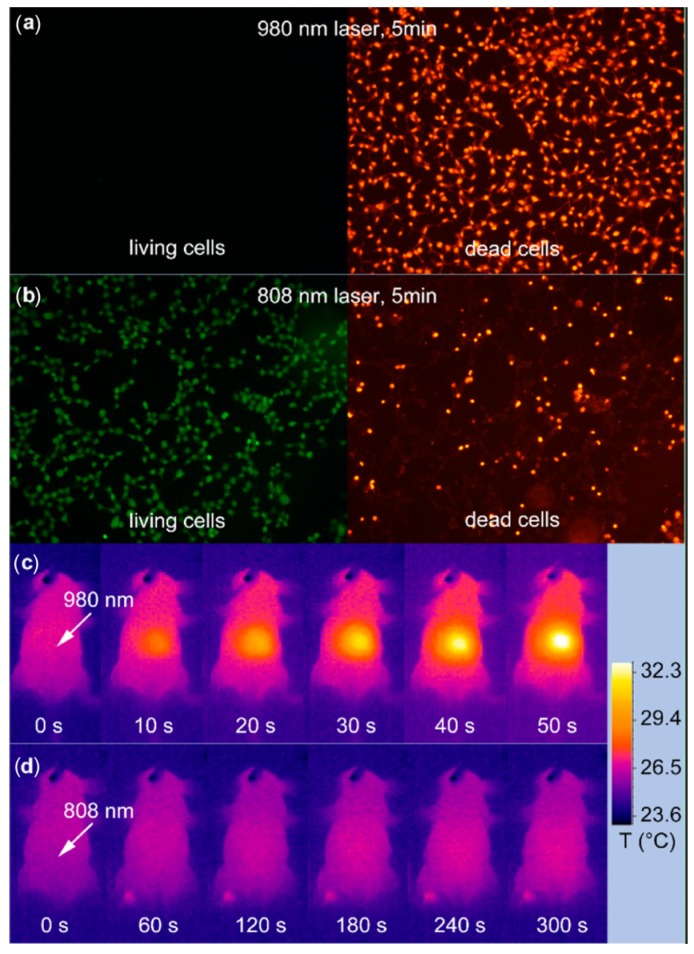
*In vitro* and *in vivo* heating effect induced by laser irradiation. (**a**,**b**) HEK (Human Embryonic Kidney) 293T cells after 5 min irradiation of 980 nm (**a**) and a 808 nm laser (**b**). Living cells and dead cells were stained with calcein AM (Acetomethoxy) and propidium iodide, respectively. (**c**,**d**) Infrared thermal image of a nude mouse during continuous (**c**) 980 nm laser irradiation for 50 s and (**d**) 808 nm laser irradiation for 300 s. Irradiation spots are denoted with the white arrows. Adapted with permission from reference [113]. Copyright: American Chemical Society, 2013.

For example, one major challenge of the utilities of UCNPs is to develop new classes of nano-materials with higher stability and converting optical properties under relatively shorter wavelength light illumination. Currently, most of the irradiation wavelength used for the excitation of the existing UCNPs systems relies on NIR laser at 980 nm, which overlaps the absorption band of the water molecule, one of the most abundant and significant NIR absorber in living systems [82]. Therefore, the 980 nm light irradiation for exciting UCNPs luminescence would be significantly attenuated in the process of penetrating biological tissues. Moreover, upon overexposure with 980 nm irradiation, the biological tissues would easily undergo overheating, and could be potentially damaged by the inevitable thermal effects [74,113] which may greatly limit their extensive applications in living systems. Therefore, new types of UCNP materials with light irradiation at shorter wavelengths would be promising alternatives to overcome the overheating effect [113] (Figure 12). Toward this direction, recently, Hummelen *et al*. [114] reported that some dye-sensitized UCNPs could be excited by 800 nm light irradiation when the lanthanide based UCNPs materials were doped with Nd. Similarly, Han *et al*. [115] also independently presented a new generation of Nd^3+^/Yb^3+^/Er^3+^ (Tm^3+^) cascade sensitized tri-doped core/shell β-NaYF_4_ colloidal UCNPs with a constitutional excitation peak at 800 nm. With a similar design, core-shell UCNPs with doping of Nd^3+^ were also reported, which were found to be effectively excited at 795 nm [116]. The cellular experiments demonstrated that the Nd^3+^-doped UCNPs with 795 nm irradiation indicated better biocompatibility compared with the conventional UCNPs under 980 nm laser irradiation. Very recently, Yan *et al*. [110] reported a new set of Nd^3+^-sensitized upconversion nanophosphors by introducing Nd^3+^ as the sensitizer to build up the core/shell structure to ensure successive Nd^3+^→Yb^3+^→activator energy transfer. The *in vivo* imaging showed that 808 nm laser excitation would be efficient for *in vivo* imaging applications. Meanwhile, the established Er@Nd UCNPs and Tm@Nd UCNPs could indicate promising multiplexing imaging effects upon laser irradiation at 808 nm. The *in vivo* overheating effect experiments confirmed that the 808 nm laser-induced local overheating effect could be greatly minimized when compared with that of the 980 nm laser (Figure 13).

In terms of the efforts to minimize the overheating effects caused by long wavelength laser irradiation, the biocompatibility and potential long-term toxicity of UCNPs would be other concerns to be addressed. Although emerging experimental results showed negligible cytotoxicity *in vitro* and *in vivo* when the particle surface was modified with biocompatible ligand moieties, longer time effects for immune response or the reproductive system still require further investigation. Furthermore, the physicochemical properties of UCNPs regarding their surface functionalization, shape, size distribution and their further functions towards reticuloendothelial system (RES) uptake such as in liver, spleen, *etc*., would also be factors to potentially influence their extensive biomedical applications in living systems.

Last, but not least, despite the fact that the UCNP particles have provided unique optical properties that make them successful for various imaging applications in biological systems, the development of alternative strategies or new sets of functional UCNPs with higher converting efficiency, enhanced photostability and quantum yields still remains a big challenge in the related fields. For instance, most existing UCNPs heavily rely on the key component of NaYF_4_ as the most efficient host materials. The significant enhancement of upconversion luminescence (UCL) efficiency is still difficult to be further achieved. Although some near-infrared dye molecules have been chosen recently as antennas to broaden and enhance the excitation of UCNPs, which could greatly improve the conversion efficiency (e.g., >3000 times) due to the increased absorptivity of the particle complexes [117], new host functional materials with more intense UCL properties to replace NaYF_4_ are still highly desired. Furthermore, the suitable size distribution of the UCNPs would be another factor to greatly affect the efficiency of upconversion luminescence. It is known that a smaller size of UCNPs would lead to a weaker luminescence emission, although smaller sized nanoparticles may indicate obvious advantages to facilitate the effective cellular or tissue uptake in the process of imaging applications. However, unfortunately, there is still a lack of satisfactory strategies to prepare the well-defined sub-10 nm UCNPs with a potent luminescence emission so far, which remains an important area with extensive efforts desperately required. Therefore, in the near future, new synthetic methods and alternative functional UCNP materials with more unique optical properties, a higher quantum yield, improved efficiency and a promising excitation wavelength will continue to be developed, which could significantly address the potential issues observed in most of the currently existing lanthanide-based UCNPs. With more innovations in material chemistry, photo-physics and biology, functional UCNPs nanostructures will continually expand their applications, and they will undoubtedly supply more exciting new insights into bio-sensing, non-invasive molecular imaging, effective theranostics, targeted drug or gene delivery and light-controlled personal diseases treatments.
